# Development and validation of a dynamic nomogram for predicting enteral nutrition feeding intolerance in patients with severe traumatic brain injury: An external validation study

**DOI:** 10.1097/MD.0000000000042993

**Published:** 2025-06-27

**Authors:** Yibo Pan, Chen Min, Jing Li, Wang Lin, Baiyu Li, Yuchao Wang, Shenhao Zhang, Wang Jing, Xiangguo Dang

**Affiliations:** a Department of Coronary Care Unit, The First Affiliated Hospital of Shandong First Medical University & Shandong Provincial Qianfoshan Hospital, Jinan, China; b School of Nursing, Shandong First Medical University, Jinan, China; c Department of Neurosurgical Intensive Care Unit, The Second Affiliated Hospital of Shandong First Medical University, Taian, China; d Department of Public Health and Preventive Medicine, The Second Affiliated Hospital of Shandong First Medical University, Taian, China; e Department of General Surgery, The Second Affiliated Hospital of Shandong First Medical University, Taian, China.

**Keywords:** dynamic nomogram, enteral nutrition feeding intolerance, prediction model, severe traumatic brain injury

## Abstract

This study aims to develop and validate a risk prediction model for enteral nutrition feeding intolerance (ENFI) in patients with severe traumatic brain injury (STBI), providing a foundation for the prevention and management of ENFI in this population. STBI is a prevalent acute and severe condition encountered in neurosurgery. STBI patients are prone to diarrhea, reflux, and other manifestations of feeding intolerance during enteral nutrition, which not only affects the patient’s systemic therapy and prolongs hospital stay, but also increases the risk of infection. We conducted a retrospective cohort study. Clinical assessment and data collection were obtained through an electronic medical record system. Data were collected from January 2019 to July 2023, we conducted a retrospective analysis of patients with STBI who met the inclusion criteria but did not meet the exclusion criteria, formed the development cohort and validation cohort. The dynamic nomogram was constructed and validated in R software. A total of 302 patients in the development cohort and 107 patients in the validation cohort were included, with incidences of ENFI at 50.7% and 56.1%, respectively. We developed a dynamic nomogram in patients with STBI and the mean arterial pressure, mechanical ventilation, intake and output, and combined antibiotics were independent predictors of ENFI. The C-index and the Hosmer–Lemeshow indicated good calibration; The calibration curve showed strong consistency between actual and predicted outcomes. The decision curve analysis confirmed the model’s clinical utility. The prediction of enteral feeding intolerance can be conveniently facilitated by the ENFI which integrates general information, condition monitoring, and therapeutic factors in patients with STBI. Based on the dynamic nomogram, medical and nursing staff in the intensive care unit can assess patients at high risk for ENFI at an early stage. This has the potential to prevent the occurrence of ENFI and enhance various clinical outcomes for patients.

## 1. Introduction

Severe traumatic brain injury (STBI) refers to patients with brain injury who have been subjected to direct or indirect trauma to the head, leading to a coma lasting longer than 6 hours, or a deterioration in consciousness within 24 hours post-injury, followed again by a coma of over 6 hours, with a Glasgow Coma Scale (GCS) score of ≤8, a prevalent acute and severe condition encountered in neurosurgery.^[1,2]^ According to experts' consensus, most critical patients without contraindications should receive enteral nutrition support within 24 to 48 hours after admission.^[3,4]^ However, STBI patients are particularly susceptible to gastrointestinal intolerance manifestations such as reflux, diarrhea, delayed gastric emptying, gastric retention, etc, during enteral nutrition due to gastrointestinal dysmotility and intestinal malabsorption.^[5]^ Enteral nutrition feeding intolerance (ENFI) is defined as the presence of high gastric residual volume (GRV) along with other gastrointestinal signs and symptoms, including vomiting, reflux, and diarrhea, which necessitate the suspension or discontinuation of enteral feedings,^[6]^ leading to the energy intake of <83.72 KJ/(kg/d) within 72 hours after the initiation of enteral nutrition. Related literature reported^[7–9]^ that the incidence of ENFI in intensive care unit (ICU) patients ranges from 33.3 to 51%, while in STBI patients with poor prognoses, this incidence rises to 59.9%. This elevated risk of ENFI is attributed to disruption in the bidirectional neural pathways between the brain and the gastrointestinal tract, known as the brain-gut axis, which can lead to gastrointestinal dysfunction in neurological disorders such as traumatic brain injury.^[10,11]^ ENFI not only complicates the overall treatment plan, extending ICU stays and increasing the risk of infection,^[12–14]^ but also hinders the nutritional supply of patients with STBI, leading to feeding difficulties, prolonged inadequate nutritional intake, and a heightened risk of malnutrition, which can delay recovery and adversely affect patient prognosis.^[15]^ Therefore, ENFI is a critical concern in the implementation of enteral nutrition for STBI patients. In this study, we constructed a risk prediction model of ENFI in patients with STBI based on R software, aimed at assisting clinical staff in individualizing the prediction and early prevention of ENFI, thereby reducing its incidence and advancing the management of ENFI prevention and treatment.

## 2. Methods

### 2.1. Study design and participants

In the development cohort, a retrospective research approach was employed to screen all patients with STBI admitted to the Neurosurgical ICU of a class A tertiary hospital in Shandong Province from January 2019 to July 2023. For the validation cohort, a similar retrospective study was conducted, including STBI patients admitted to the Neurosurgical ICU of another Class A tertiary hospital in Shandong Province from January 2022 to July 2023, following the same inclusion and exclusion criteria as the development cohort. Inclusion criteria: patients admitted to the Neurosurgical ICU, who met the diagnostic criteria for STBI,^[16]^ with a clear history of traumatic brain injury and confirmation of cranial injury through MRI and/or CT examination; GCS score of 3 to 8; no preexisting organ pathology or nutritional, immune, and metabolic disorders prior to the injury; no concurrent abdominal injuries or gastrointestinal dysfunction; enteral nutritional support provided via nasogastric or nasoenteric tubes. Exclusion criteria: patients who had received enteral nutrition prior to admission to the neurosurgical ICU; neurosurgical ICU stays <5 days and/or enteral nutrition for fewer than 3 days for reasons unrelated to ENFI; pregnant or postpartum patients; age < 18 years.

### 2.2. Sample size consideration

Referring to the sample size estimation method for multivariate logistic regression analysis, the sample size should be at least 5 to 10 times the number of study variables.^[17]^ In this study, 19 independent variables were explored and the prevalence of ENFI in patients with STBI was found to be 59.9%.^[9]^ This is a retrospective study with potential for missing data, considering a sample attrition rate of 10%. Therefore the study required a minimum of 174 to 349 patients for modeling.

### 2.3. Endpoint

ENFI is defined as the interruption or suspension of enteral nutrition due to gastrointestinal adverse effects such as diarrhea, bloating, vomiting or reflux, high GRV, etc, occurring during enteral nutrition,^[6]^ which results in the patient’s energy intake being insufficient to 83.72 KJ/(kg/d) within 72 hours of initiating enteral nutrition. ENFI diagnostic criteria, as proposed by the European Society of Critical Care Medicine regarding abdominal issues^[18]^:

1. Diarrhea: At least 3 fluid or semi-fluid stools or defecation exceeding 250 mL per instance within 24 hours;
2. Abdominal distension: Manifestations of abdominal distension, abdominal muscle tension, tympanic sound on percussion, signs of intestinal pneumatosis or effusion on X-ray, and monitoring of elevated intra-abdominal pressure (IAP) (2 consecutive measurements within 6 h, both IAP > 12mmHg);
3. Vomiting/reflux: Presence of gastric contents in the mouth, regardless of the amount, or nutrient fluids flowing from the nasogastric or nasoenteric tube;
4. High GRV: For continuous enteral nutrition infusion with syringe aspiration of GRV every 6 hours. Recorded as slowing of enteral nutrition rate or suspension of enteral nutrition due to high GRV.

### 2.4. Instrument

The questionnaire on factors influencing ENFI in patients with STBI, which was developed based on research variables identified through literature review and expert panel discussion. It comprised 3 sections: General information (age, gender, etc); Condition monitoring (blood glucose, GCS, blood potassium, etc); Therapeutic factors (enteral nutrient composition, combined parenteral nutrition, subcryotherapy, etc).

By synthesizing definitions and assessment methods for ENFI from the literature and recommendations from the expert panel, researchers ultimately developed the ENFI assessment form for patients with STBI. The indicators of ENFI included 4 items, including diarrhea, vomiting/reflux, abdominal distension, and high GRV. These indicators were monitored for 5 consecutive days following the initiation of enteral nutrition, as studies have shown that the average time from the start of enteral nutrition to the occurrence of ENFI is approximately 3 days.^[19]^ The occurrence of any of the 4 indicators during the observation period was considered indicative of ENFI.

### 2.5. Data collection

The researcher utilized the electronic medical record system (hospital information system) to collect relevant patient information in accordance with the questionnaire on factors influencing ENFI in patients with STBI. Recording the general information of the patients, such as gender, age, primary diagnosis, therapeutic factors, and indicators of condition monitoring for 5 consecutive days following the initiation of enteral nutrition. The researcher collected relevant biochemical indicators, GCS scores, temperature, and mean arterial pressure (MAP), recording the worst indicator values within the 24 hours prior to assessment. For the indicators that were not examined on the day of the assessment, the results of the most recent examination were recorded, and the intake and output over 24 hours were recorded (intake and output = total intake-total output, intake, and output > 0 for positive equilibrium, intake, and output < 0 for negative equilibrium) Therapeutic factors were obtained from the intensive care orders for that day. The occurrence of ENFI was determined based on the enteral nutrition tolerance assessment form; if ENFI occurred, data collection for that patient was concluded; if ENFI did not occur, observation continued until the medical records were collected for 5 days.

### 2.6. Statistical analysis

The statistical analysis and graphics were conducted by SPSS version 25.0 (IBM SPSS Statistics, Armonk, New York) and R version 4..2.2 (R Foundation for Statistical Computing, Vienna, Austria), statistical significance was defined as *P* < .05. The normal distribution of continuous variables was assessed using the Shapiro–Wilk test. For skewed distributions, the Wilcoxon–Mann–Whitney *U* test was employed and results were presented as the median and interquartile range. Categorical variables were described using frequency (percentage) and compared using either the Pearson chi-squared test or Fisher exact test, depending on appropriateness. Initially, least absolute shrinkage and selection operator (LASSO) regression analysis was performed using the glmnet package, and the lambda (*λ*) values were calculated via cross-validation to screen the predictors. Subsequently, the predictors identified by LASSO regression were incorporated into a multivariate logistic regression analysis using the RMS package. Variables with *P* < .05 were selected to construct the prediction model. Ultimately, we constructed dynamic nomogram with the DynNom package. Discrimination was assessed by calculating the area under the receiver operating characteristic (ROC) curve derived from conventional ROC curves. The calibration plots of the nomogram were evaluated using the Hosmer–Lemeshow test. The discrimination and calibration of the nomogram model were validated in an independent external validation cohort. Additionally, decision curve analysis (DCA) was conducted to assess the net benefit of decision-making for ENFI using the nomogram at various threshold probabilities in the validation cohort.

### 2.7. Ethical considerations

The study had been approved by The Second Affiliated Hospital of Shandong First Medical University ethics committee. Clinical trial registration number: 2022-092. As this was a retrospective study, it was approved without requiring informed consent from participants.

## 3. Results

### 3.1. Clinical and demographic data for development and validation cohort

The cohorts were formed based on the data available from each center. A total of 302 STBI patients were included in the development cohort, with 50.7% of patients occurred ENFI. Then a total of 107 STBI patients, with 56.1% of patients occurred ENFI, were involved in the validation cohort. The baseline information of the development cohort(n = 302) and validation cohort (n = 107) was analyzed and compared, and the results showed that there were no significant differences in any variables between the 2 groups (*P* > .05). More details are presented in Table 1.

**Table 1:** Clinical and demographic data for development and validation cohort.

Variables	Category	Development cohort (n = 302)	Validation cohort (n = 107)	Statistics	*P* value
Age, yr		60.59 ± 14.14	58.66 ± 12.86	1.220*	.176
Blood glucose, mmol/L		9.80 (8.20–12.28)	9.60 (8.10–12.50)	−0.162†	.871
Albumin,g/L		36.94 ± 5.30	36.54 ± 5.20	0.680*	.369
Potassium, mmol/L		3.97 ± 0.53	3.93 ± 0.51	0.796*	.540
MAP, mm Hg		84.78 ± 14.23	86.15 ± 14.56	−0.852*	.274
Abrosia/	≤3 d	162 (53.64)	53 (49.53)	0.383‡	.536
Parenteral nutrition time	>3 d	140 (46.36)	54 (50.47)		
Gender	Male	201 (66.56)	61 (57.01)	2.727‡	.099
	Female	101 (33.44)	46 (42.99)		
EN recipe	Proteotype	221 (73.18)	73 (68.22)	0.730‡	.393
	Short peptide	81 (26.82)	34 (31.78)		
Combined PN	No	154 (50.99)	47 (43.93)	1.309‡	.253
	Yes	148 (49.01)	60 (56.07)		
Mild hypothermia	No	268 (88.74)	97 (90.65)	0.135‡	.714
	Yes	34 (11.26)	10 (9.35)		
MV	No	191 (63.25)	66 (61.68)	0.029‡	.864
	Yes	111 (36.75)	41 (38.32)		
Analgesic/ sedative	No	66 (21.85)	21 (19.63)	0.120‡	.729
	Yes	236 (78.15)	86 (80.37)		
Vasoactive drug	No	72 (23.84)	20 (18.69)	0.924‡	.336
	Yes	230 (76.16)	87 (81.31)		
Combined antibiotics	No	242 (80.13)	92 (85.98)	1.435‡	.231
	Yes	60 (19.87)	15 (14.02)		
GCS	3–5 score	174 (57.62)	72 (67.29)	2.694‡	.101
	6–8 score	128 (42.38)	35 (32.71)		
Temperature	≤38.5°C	255 (84.44)	88 (82.24)	0.142‡	.706
	>38.5°C	47 (15.56)	19 (17.76)		
Intake and output	Negative equilibrium	104 (34.44)	39 (36.45)	0.066‡	.797
	Positive equilibrium	198 (65.56)	68 (63.55)		

Variables	Category	Development cohort (n = 302)	Validation cohort (n = 107)	Statistics	*P* value
Dehydrant drug	No	83 (27.48)	27 (25.23)	0.105‡	.746
	Yes	219 (72.52)	80 (74.77)		
Potassium drug	No	189 (62.58)	66 (61.68)	0.002‡	.961
	Yes	113 (37.42)	41 (38.32)		

EN = enteral nutrition, GCS = Glasgow Coma Scale, MAP = mean arterial pressure, MV = mechanical ventilation.

* = t.

† = z.

‡ = *χ*^2^.

### 3.2. LASSO regression analysis of ENFI in patients with STBI

**Figure 1.**
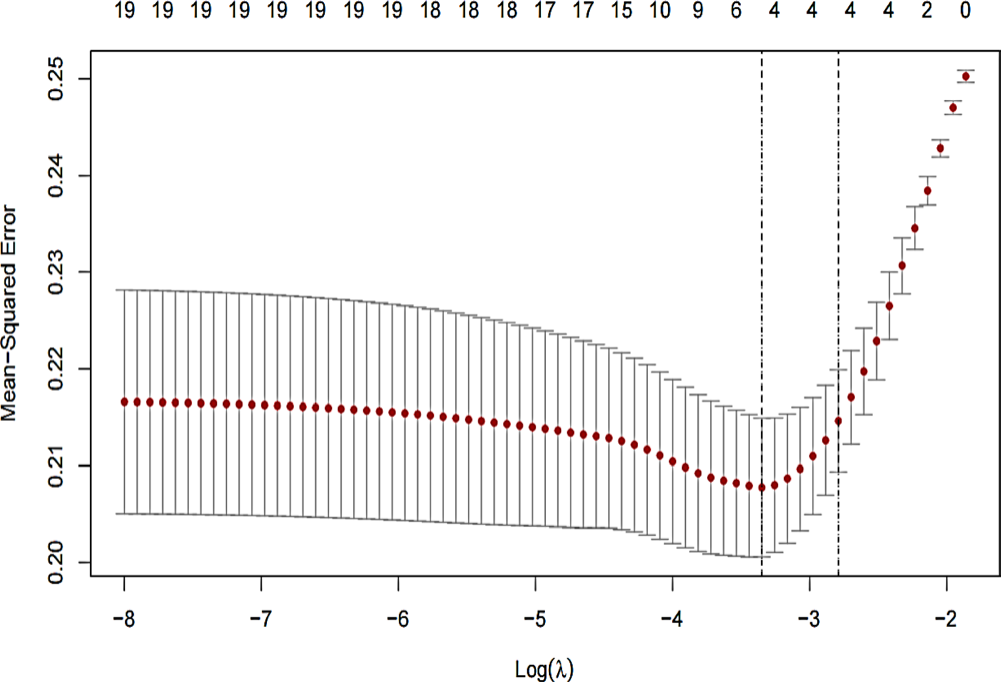
LASSO regression internal cross-validation plot. LASSO = least absolute shrinkage and selection operator.

**Figure 2.**
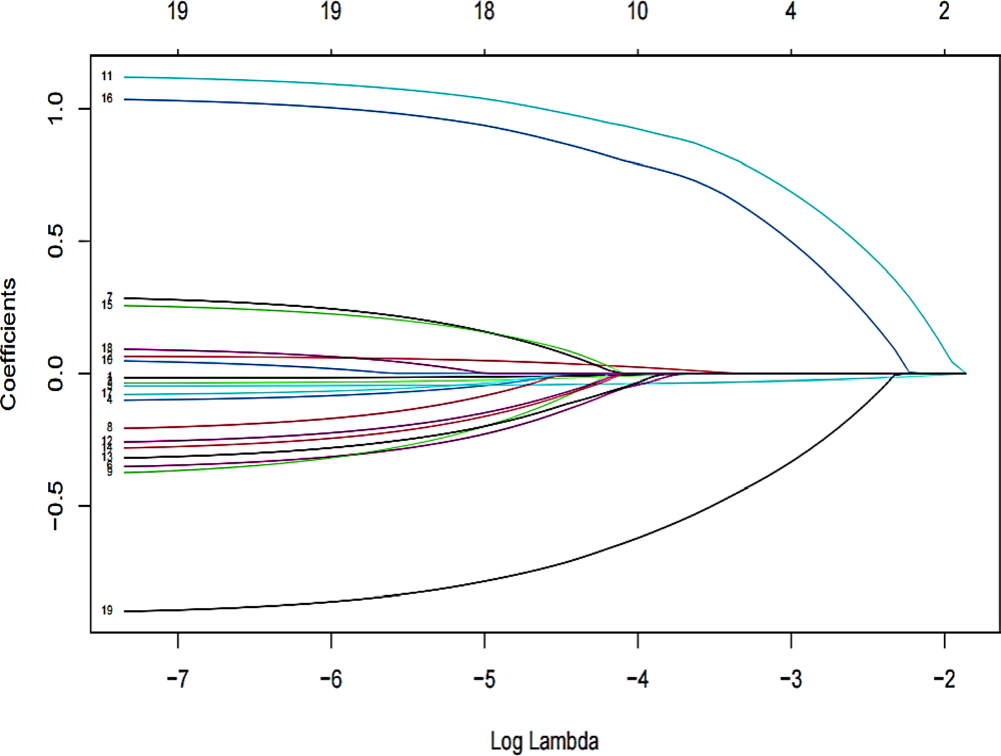
Plot of regression distribution coefficients for each predictor variable in relation to ENFI. ENFI = enteral nutrition feeding intolerance.

This study examined 19 influencing factors, previously, we employed LASSO regression to screen the variables. The 19 influencing factors were treated as independent variables, while the occurrence of ENFI served as the dependent variable. We selected the penalty parameter *λ* that minimized the biased likelihood error using 10-fold cross-validation, as shown in Figure 1. As *λ* increased, the estimated parameters were progressively reduced, and beyond a certain value of *λ*, some unimportant variables were compressed to zero, indicating their exclusion from the model. The changes in the regression coefficients for each variable are depicted in Figure 2. The λ.min corresponded to the *λ* that minimized the model’s estimation error, resulting in the selection of 5 variables: mechanical ventilation, MAP, combined antibiotics, intake and output, and blood glucose. Conversely, the λ.lse corresponded to the minimum mean squared error plus 1 standard error, yielding 4 selected variables: intake and output, mechanical ventilation, MAP, and combined antibiotics. A lower mean squared error indicates a better model, thus, we selected the *λ* with the smallest estimation error (*λ* = 0.035) as the optimal penalization coefficient for the model, which included a total of 5 variables: mechanical ventilation, MAP, combined antibiotics, intake and output, blood glucose.

### 3.3. Multivariate logistic regression analysis of ENFI in patients with STBI

We included the 5 predictors screened through LASSO regression analysis in a multivariate logistic regression analysis. The results indicated that MAP (odds ratio [OR] = 0.956, 95% confidence interval [CI]: 0.937–0.974, *P* < .001), mechanical ventilation (OR = 3.023, 95%CI: 1.758–5.199, *P* < .001), intake and output (OR = 0.441, 95%CI: 0.254–0.765, *P* = .004), and combined antibiotics (OR = 2.980, 95%CI: 1.491–5.954, *P* = .002) were statistically significant (*P* < .05), while blood glucose did not demonstrate statistical significance (*P* > .05) (Table 2).

**Table 2:** Multivariate logistic regression analysis of predictors for enteral nutrition feeding intolerance in the development cohort.

Variables	Beta coeff	SE	Ward *χ*^2^	*P* value	OR(95% CI)
constant	3.300	0.938	12.370	<.001	27.114
MAP	−0.045	0.010	20.881	<.001	0.956 (0.937–0.974)
MV	1.106	0.277	15.993	<.001	3.023 (1.758–5.199)
Combined antibiotics	1.092	0.353	9.558	.002	2.980 (1.491–5.954)
Intake and output	−0.819	0.281	8.496	.004	0.441 (0.254–0.765)
Blood glucose	0.048	0.036	1.787	.181	1.049 (0.978–1.125)

Beta coeff = Beta coefficient, CI = confidence interval, MAP = mean arterial pressure, MV = mechanical ventilation, OR = odds ratio.

### 3.4. The dynamic nomogram of ENFI in patients with STBI

**Figure 3.**
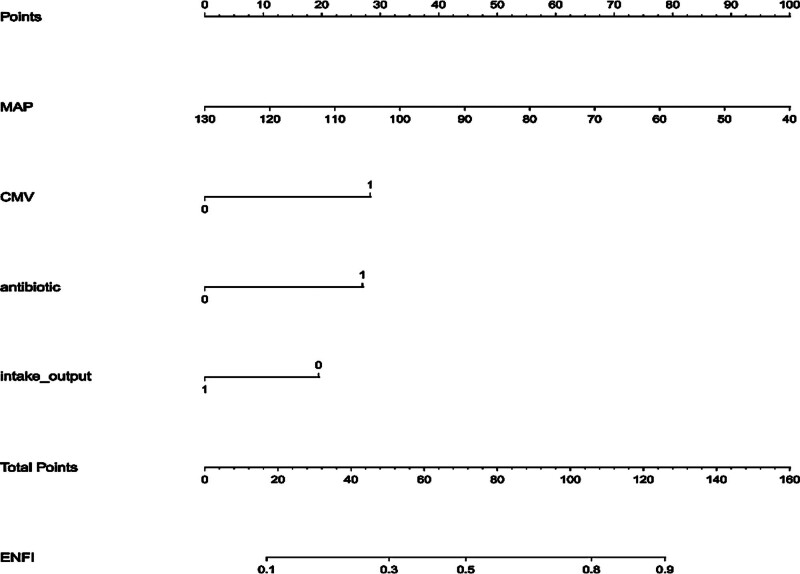
Nomogram of ENFI in patients with STBI. ENFI = enteral nutrition feeding intolerance, STBI = severe traumatic brain injury.

Based on the results of multivariate logistic regression analyses, we constructed a nomogram containing 5 independent predictors for the risk of ENFI in patients with STBI (Fig. 3). The value of each factor was assigned a score on a point scale axis. The total score can be easily calculated by adding up each individual score, and then by projecting the total score onto the bottom risk scale axis, we can estimate the probability of ENFI. Also, to facilitate clinical practice, we constructed a dynamic web-based online nomogram, which can be accessed at https://enfi.shinyapps.io/stbienfi/.

### 3.5. Validation of the nomogram model for ENFI in patients with STBI

**Figure 4.**
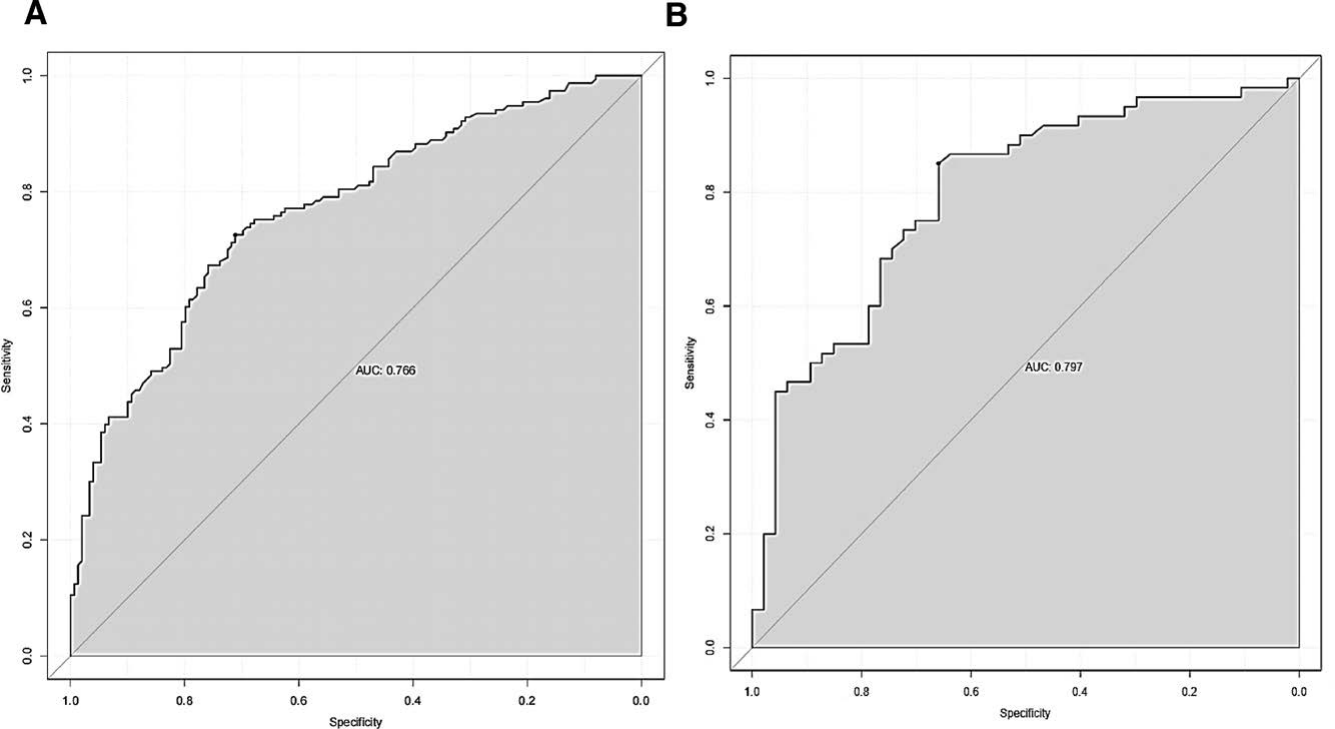
(A) The ROC curve of the nomogram in the development cohort. (B) The ROC curve of the nomogram in the validation cohort. AUC = area under the receiver operating characteristic curve, ROC = receiver operating characteristic.

**Figure 5.**
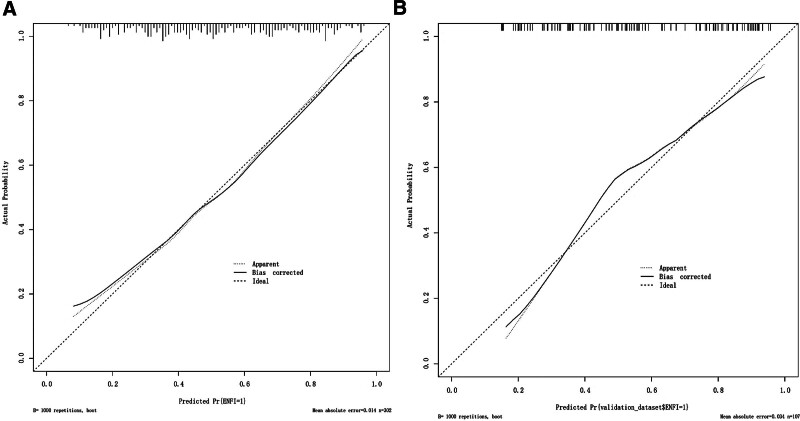
(A) The calibration plot for nomogram in the development cohort. (B) The calibration plot for nomogram in the validation cohort.

In the development cohort (Fig. 4A), the nomogram model achieved an area under the receiver operating characteristic curve (AUC) of 0.766 (95% CI: 0.714–0.819), while the AUC of the nomogram model in validation cohort (Fig. 4B) was 0.797 (95% CI: 0.711–0.882). Calibration diagrams revealed that the actual prediction curve and the simulated prediction curve exhibited a similar trend in both the development and validation cohort (Fig. 5A and B), indicating strong calibration and high predictive accuracy. The Hosmer–Lemeshow test demonstrated no significant miscalibration in the 2 datasets (*P* = .807 in the development cohort and *P* = .966 in the validation cohort), suggesting a well-fitted model.

### 3.6. Clinical use

**Figure 6.**
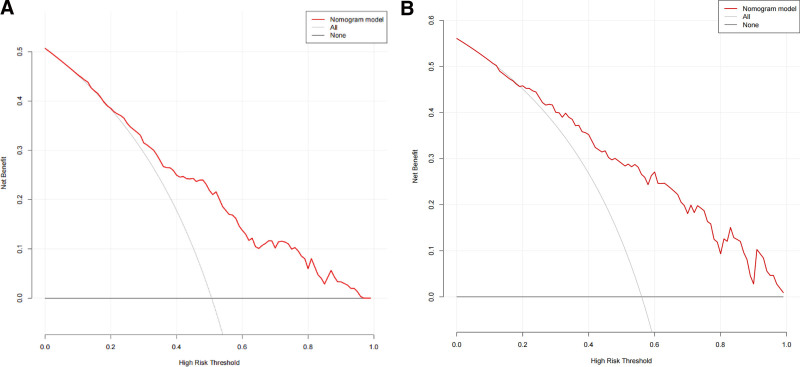
(A) Decision curve analysis for nomogram in the development cohort. (B) Decision curve analysis for nomogram in the validation cohort.

DCA curves were plotted for the occurrence of ENFI in patients with STBI in the development cohort and the validation cohort respectively(Fig. 6A and B). The Y-axis represents the net benefit, while the X-axis represents the threshold probability of ENFI occurrence that medical staffs consider likely. The “All” line represents the scenario where all patients receive intervention, while the “None” line represents the scenario in which no patients receive intervention, resulting in a net benefit of 0. The “Nomogram model” curve reflects the specific decision curve associated with the present study. The DCA curves of both the development cohort and the validation cohort showed that the net benefit of the model constructed in this study was higher, indicating that the model has good clinical benefits and some clinical application value.

## 4. Discussion

### 4.1. The nomogram model has better predictive efficacy

ROC analysis was performed on the predictors of ENFI occurrence in both the development and validation cohort. The AUCs (95% CI) were 0.766 (95% CI: 0.714–0.819) for the development cohort and 0.797 (95% CI: 0.711–0.882) for the validation cohort, confirming the favorable discriminative ability of the nomogram model. In this study, the Hosmer–Lemeshow test combined with calibration curve was utilized to assess the accuracy of the nomogram in predicting the probability of ENFI in patients with STBI. The results indicated that in the development cohort, *χ*^2^ = 4.521, *P* = .807, and in the validation cohort, *χ*^2^ = 2.403, *P* = .966, with both *P* > .05, suggesting that the difference between the actual and predicted outcomes was not statistically significant. To further assess the calibration performance of the nomogram model, calibration diagrams were constructed for both cohorts. The x-axis represented the predicted risk of ENFI occurrence, while the y-axis represented the actual risk of ENFI occurrence. The diagonal dotted lines represented prediction models with perfect predictive power. A closer alignment between the calibration curve and the diagonal dashed line indicated higher prediction accuracy of the nomogram model. Notably, both curves exhibited slight linearity, indicating excellent calibration performance of the model. In developing the risk prediction model, it was inevitable to encounter the problem of false positives and false negatives. Medical staff determined whether to intervene based on the model’s predictions, as these inaccuracies can affect the net benefit rate of the prediction model. To address this limitation, this study introduced the DCA curve to evaluate the prediction model. Net benefit was calculated by subtracting the proportion of false-positive patients from the proportion of true positives, weighted based on the relative harm of withholding treatment versus the negative consequences of unnecessary treatment. The threshold probability signified the likelihood of ENFI occurrence, guiding critical care surgeons in deciding whether to delay EN based on this probability. The DCA curve indicated that the threshold probability ranged from 20 to 99%, with the model demonstrating a net benefit >0. Within this range, providing appropriate interventions was more beneficial than either the patient “receiving all interventions” or “receiving no interventions.” Moreover, across a wider threshold range, the net benefit remained positive, indicating that the nomogram model possessed high clinical applicability and ensured accuracy within a broad safety margin. Consequently, a nomogram model characterized by high discrimination, calibration, and clinical utility was developed to predict the probability of ENFI in patients with STBI.

### 4.2. Influencing factors of ENFI in patients with STBI

#### 4.2.1. Effect of combined antibiotics on ENFI in patients with STBI

This study confirmed that combined antibiotics was an independent risk factor for ENFI in patients with STBI. Due to the reduced autoimmune function in STBI patients, surgeons may increase the type or dosage of antibiotics to prevent infectious diseases, leading to deterioration of the condition. While antibiotics combat pathogenic bacteria, they can also disrupt the normal intestinal flora, leading to an imbalance.^[20]^ Multivariate logistic regression analysis in this study confirmed that combined antibiotics (OR = 2.980, 95%CI: 1.491–5.954, *P* = .002) increased the incidence of ENFI in patients with STBI, a finding corroborated by related studies.^[21]^ A survey showed that most ICU nurses may overlook the administration of antibiotics due to their heavy workload.^[22]^ Therefore, enhancing nurses’ knowledge about antibiotics through training is essential to ensure proper indications, standardize usage, understand the half-lives of antibiotics, and establish appropriate administration intervals.^[23]^ For patients requiring combined antibiotic therapy, the addition of probiotics may serve as a preventive measure. A study confirmed^[24]^ that probiotics can reduce the incidence of gastrointestinal complications, improve gastrointestinal function, and decrease the incidence of ENFI in patients with enteral nutrition.

#### 4.2.2. Effect of mechanical ventilation on ENFI in patients with STBI

Several studies have confirmed that mechanical ventilation increased the incidence of ENFI,^[25,26]^ which was consistent with the findings of this study. The reason for this may be that high levels of positive end-expiratory pressure can reduce gastrointestinal mucosal blood perfusion, leading to gastrointestinal ischemia and hypoxia. In addition, elevated inferior vena cava pressure also increases the resistance to gastrointestinal perfusion, causing gastrointestinal mucosal damage and weakened gastrointestinal function.^[27]^ Furthermore, the phenomenon of patient-ventilator asynchrony can occur during mechanical ventilation, leading to increased oxygen consumption and further gastrointestinal dysfunction, which raises the risk of complications such as gastric retention and flatulence.^[28]^ Multivariate logistic regression analysis showed that mechanical ventilation (OR = 3.023, 95%CI: 1.758–5.199, *P* < .001) was an independent risk factor for ENFI in patients with STBI. Consequently, clinical staff should remain vigilant when managing enteral nutrition in mechanically ventilated patients, dynamically assessing their gastrointestinal functional status. Enteral nutrition support strategies should be formulated to anticipate and prevent ENFI, such as implementation of post-pyloric feeding. Evidence suggested that enteral nutrition via jejunal tube was safe and effective in critically ill patients, demonstrating superior tolerance and reduced reflux rates compared to gastric tubes.^[29]^ A meta-analysis^[30]^ showed that patients receiving post-pyloric feeding had a lower incidence of enteral nutrition complications, significantly improved nutritional status, and better clinical outcomes compared to those fed via nasogastric tubes.

#### 4.2.3. Effect of MAP on ENFI in patients with STBI

MAP serves as an indicator of the body’s circulating blood volume and tissue perfusion, and the study showed that MAP (OR = 0.956, 95%CI: 0.937–0.974, *P* < .001) was an independent risk factor for ENFI in patients with STBI. Notably, 1 study^[31]^ reported that gastrointestinal tolerance during enteral nutrition in hemodynamically stable critically ill patients was high. Early enteral nutrition has been shown to enhance circulatory function and ameliorate oxygen metabolism disorders, thus facilitating the restoration of intestinal function in postoperative STBI patients. However, in instances of shock, the effective circulating blood volume decreases. To preserve the functionality of vital organs such as the heart and brain, the body redistributes blood flow, consequently diminishing the blood supply to the gastrointestinal mucosa. This sustained hypoperfusion can lead to mucosal congestion, edema, compromised intestinal barrier integrity, and a shift in intestinal flora, which may exacerbate infections and further impair gastrointestinal function.^[32]^ A randomized trial demonstrated that in hemodynamically unstable patients, early enteral nutrition was associated with an increased incidence of gastrointestinal complications compared to isocaloric parenteral nutrition.^[33]^ Therefore, during enteral nutritional support therapy for STBI patients, it is crucial to closely monitor fluctuations in MAP. For those who are hemodynamically unstable, effective therapeutic measures should be implemented to maintain adequate gastrointestinal perfusion. During enteral nutritional therapy, it is advisable to adopt a gradual and stepwise approach to enhance tolerance to enteral nutrition.

#### 4.2.4. Effect of intake and output on ENFI in patients with STBI

The study showed that intake and output (OR = 0.441, 95%CI: 0.254–0.765, *P* = .004) was an independent risk factor for ENFI in patients with STBI. Fluid therapy is an important part of the treatment regimen for patients with STBI, large volumes of rehydration fluids can rapidly and effectively replenish water and electrolytes to maintain the stability of the internal environment, in addition, patients are unable to regenerate a sufficient volume of blood in a short period of time due to the blood loss state, rehydration fluids can increase the blood volume, correct the acidosis, and improve shock conditions. However, excessive rehydration may lead to cerebral edema and exacerbate intracranial hypertension. Intake and output are important indicators that clinical staff should monitor closely, and in recent years, several studies have reached varying conclusions regarding the impact of intake and output on ENFI. Atasever et al^[34]^ confirmed that patients with a negative equilibrium had a high risk of ENFI, which aligned with the findings of this study. This may be explained by the fact that an excessively negative equilibrium can place the gastrointestinal mucosa in a state of low perfusion, leading to tissue ischemia and hypoxia, which can cause manifestations of gastrointestinal intolerance. In contrast, a retrospective study found^[35]^ that negative equilibrium could reduce IAP, resulting in a lower incidence of abdominal distension and gastric retention in patients with a negative equilibrium compared to those with a positive equilibrium. Furthermore, a meta-analysis^[36]^ indicated that a positive equilibrium was associated with intra-abdominal hypertension and gastrointestinal complications. As IAP increases, intestinal mucosal edema may occur, leading to gastrointestinal dysfunction, which in turn increases the probability of ENFI. Therefore, when caring for STBI patients on enteral nutrition, clinical staff should pay attention to the intake and output and be alert to the occurrence of ENFI. Currently, there are limited studies examining the effect of intake and output on ENFI, and the underlying mechanisms warrant further research and investigation.

### 4.3. Limitations

The demographics of the participants, primarily recruited from class A tertiary hospitals, which may limit the generalizability. Furthermore, despite surpassing the calculated minimum sample size required, the number of patients included remains relatively small, which may impact the accuracy of the predictive model. Therefore, it is necessary to validate the findings of this study through larger-scale, multicenter research.

## 5. Conclusions

In conclusion, ENFI is a common complication during enteral nutrition in patients with STBI. The dynamic nomogram constructed in this study has good predictive efficacy and is convenient for clinical application, which aids in the early identification of high-risk ENFI patients and serves as a reference for advancing informatization in the prevention and treatment of ENFI.

## Acknowledgments

The authors gratefully acknowledge the administrators from the collaborating hospitals for supporting this investigation.

## Author contributions

**Data curation:** Yibo Pan, Wang Lin.

**Conceptualization:** Jing Li, Wang Lin.

**Formal analysis:** Yuchao Wang.

**Funding acquisition:** Xiangguo Dang.

**Investigation:** Yuchao Wang, Shenhao Zhang.

**Methodology:** Chen Min, Jing Li.

**Project administration:** Chen Min, Xiangguo Dang.

**Resources:** Chen Min, Shenhao Zhang, Wang Jing.

**Software:** Baiyu Li.

**Supervision:** Jing Li, Wang Jing, Xiangguo Dang.

**Validation:** Wang Lin, Baiyu Li.

**Writing – original draft:** Yibo Pan.

**Writing – review & editing:** Wang Jing, Xiangguo Dang.
